# Genome Sequences of Murine Pneumotropic Virus (*Polyomaviridae*) Detected in Wild House Mice (*Mus musculus*)

**DOI:** 10.1128/genomeA.01545-15

**Published:** 2016-01-21

**Authors:** Nicole Ben Salem, Ugo Moens, Bernhard Ehlers

**Affiliations:** a Division 12 “Measles, Mumps, Rubella and Viruses Affecting Immunocompromised Patients,” Robert Koch Institute, Berlin, Germany; b Faculty of Health Sciences, Department of Medical Biology, University of Tromsø, Tromsø, Norway

## Abstract

Using generic PCR, we identified a variant of murine pneumotropic virus (MptV) (family *Polyomaviridae*) in 3 wild house mice (*Mus musculus*). The fully amplified and sequenced genomes display considerable differences from the MptV genomes published previously and enlighten us on the natural diversity of rodent polyomaviruses.

## GENOME ANNOUNCEMENT

Two polyomaviruses (PyVs) have been identified in mice, mouse PyV (MPyV) (1) and murine pneumotropic virus (MptV). Previously designated Kilham or K virus, MptV was first identified in 1952 in laboratory mice (C3H strain) (2). Infection with MptV causes fatal interstitial pneumonia in newborn mice; in older animals, it persists inapparently (3). The MptV genome was fully sequenced from cloned DNA deposited in the American Type Culture Collection (ATCC) as clone pKV(37-1) (4), GenBank accession number NC_001505. Of note, another MptV genome was deposited in 2007 in GenBank (accession no. EF186666) and was indicated as originating from the same ATCC-deposited material, but it has not been published. The two genomes differ in their nucleotides by 8%.

Here, we report the detection of MptV in spleen (but not lung) samples from 3/4 cadaveric wild house mice (*Mus musculus*) collected in 2008 during pest control measurements in the Berlin zoological gardens. Initially, partial VP1-encoding sequences of MptV were identified with generic PCR using an established protocol (5). Sequences of overlapping PCR fragments (0.9 to 1.3 kb) amplified with primers that had been deduced from conserved regions of the published MptV sequences were then used to compile 3 full genomes. These were named MptV #6018, #6020, and #6022. Complete identity was observed for the #6018 and #6022 genomes, and the #6020 genome exhibited a single-nucleotide exchange resulting in an exchange of Phe to Ile at position 97 in the small T antigen (STAg). Compared to the NC_001505 and EF186666 genomes, they reveal 9% and 1% nucleotide differences, respectively. Upon alignment of all five genomes with the MAFFT module of Geneious 8.7.1, the NC_001505 sequence exhibited single-nucleotide insertions or deletions at several positions in coding sequences (CDSs), which caused frameshifts in, e.g., amino acid sequences of the large T antigen (LTAg) and VP1 compared to those encoded by the EF186666 and the 3 novel MptV genomes. As this observation suggested mistakes in the NC_001505 sequence, it was excluded from subsequent sequence comparisons. In comparison to the EF186666 genome, the novel MptV genomes display nucleotide exchanges in LTAg CDSs (*n* = 31) and VP1 CDSs (*n* = 13), resulting in a single-amino acid exchange in both proteins. In addition, the proline^118^ is absent in LTAg. Only synonymous nucleotide exchanges were observed in VP2 CDSs (*n* = 5) and STAg CDSs (*n* = 5), and 1 additional nonsynonymous mutation was seen in STAg of the #6020 genome. In conclusion, the full-genome sequences of MptV naturally circulating in wild mice are presented here for the first time. Future genome analysis of MptV circulating in mice from other geographical habitats is required to fully elucidate the natural diversity of MptV.

### Nucleotide sequence accession numbers.

The complete genomes of MptV #6018, #6020, and #6022 have been deposited in GenBank under the accession numbers KT987216, KT987217, and KT987218.
